# Supplementary material for: Graphene-based sensing of oxygen transport through pulmonary membranes
Source: Nat Commun. 2020 Feb 27;11:1103. doi: 10.1038/s41467-020-14825-9 (PMC7046670; doi:10.1038/s41467-020-14825-9)
Supplement: Supplementary file 1 — Supplementary Information [file 41467_2020_14825_MOESM1_ESM.pdf]

Supplementary Information

Graphene-based sensing of oxygen transport through pulmonary membranes

Kim et al.

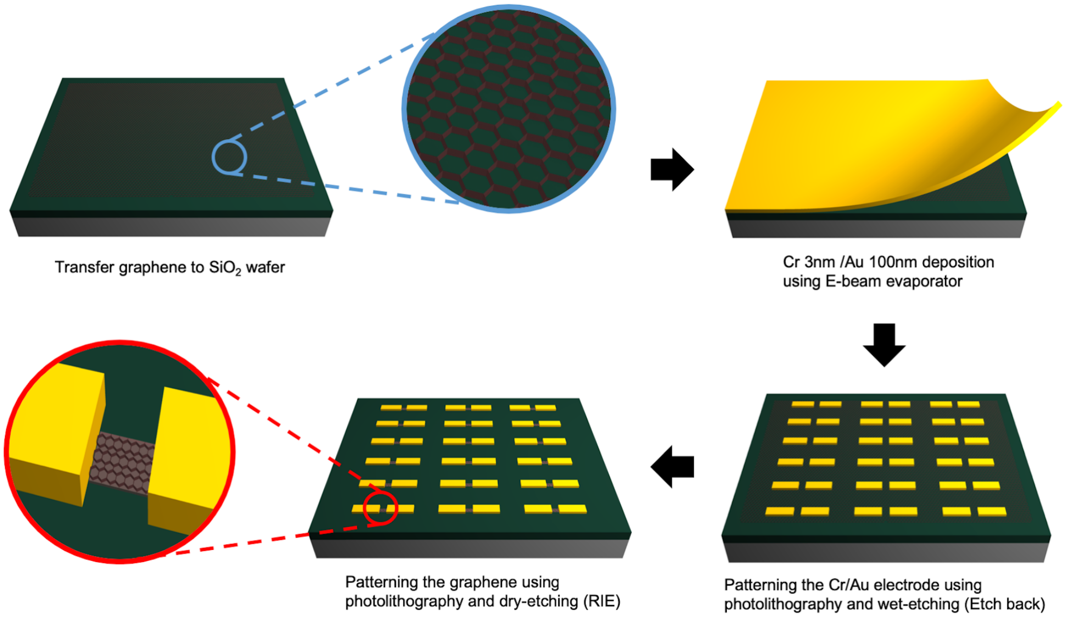

Supplementary Figure 1. Schematic representation of the assembly steps towards building the graphene-based oxygen gas sensor with patterned Cr/Au electrodes.

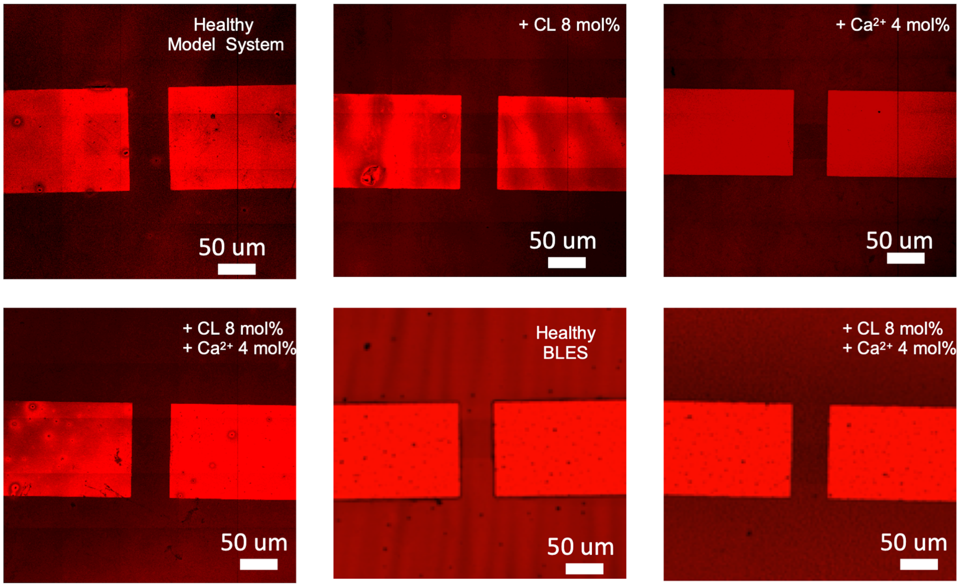

Supplementary Figure 2. Confocal fluorescence microscopy of lung membrane films tagged with Texas-Red at various compositions. The smooth films are spin coated onto the graphene sensor.

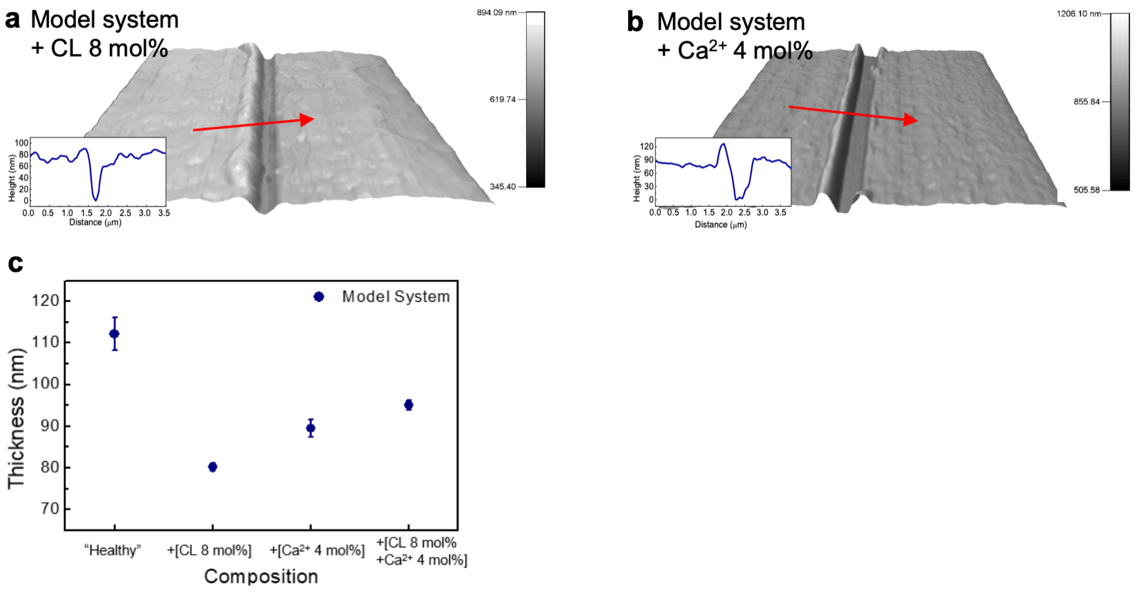

Supplementary Figure 3. AFM tip-scratch topographic images of model system containing cardiolipin (CL) (a) and CL and calcium (b) to investigate thickness of the adsorbed lipid films at different compositions. The thickness of the film is around 100 nm with a tendency to slightly thin out in diseased conditions (c). Standard deviation (n=10).

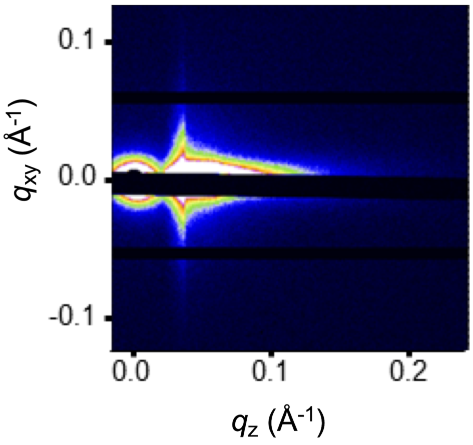

Supplementary Figure 4. 2D GISAXS data of bare graphene-based oxygen gas sensor showing no diffraction patterns other than the typical Yoneda peak and diffuse scattering halo.

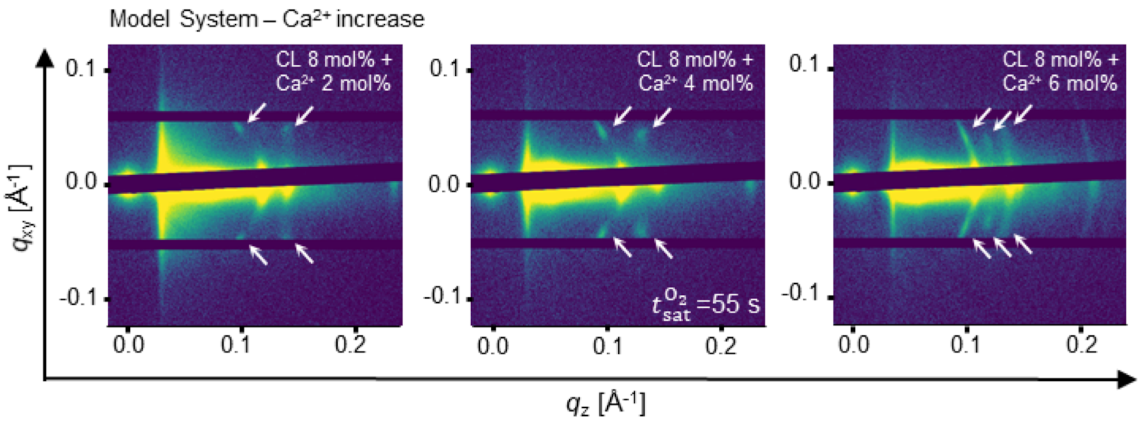

Supplementary Figure 5. 2D GISAXS data of lipid-only lung membrane model-system at fixed CL content and increasing calcium ion content. In all samples well-oriented (along q_z) multilamellar systems can be observed as well as the characteristic stalk-phase diffraction peaks (indicated by white arrows). The stalk-phase diffraction peaks become more intense and well-defined as the calcium content increases.

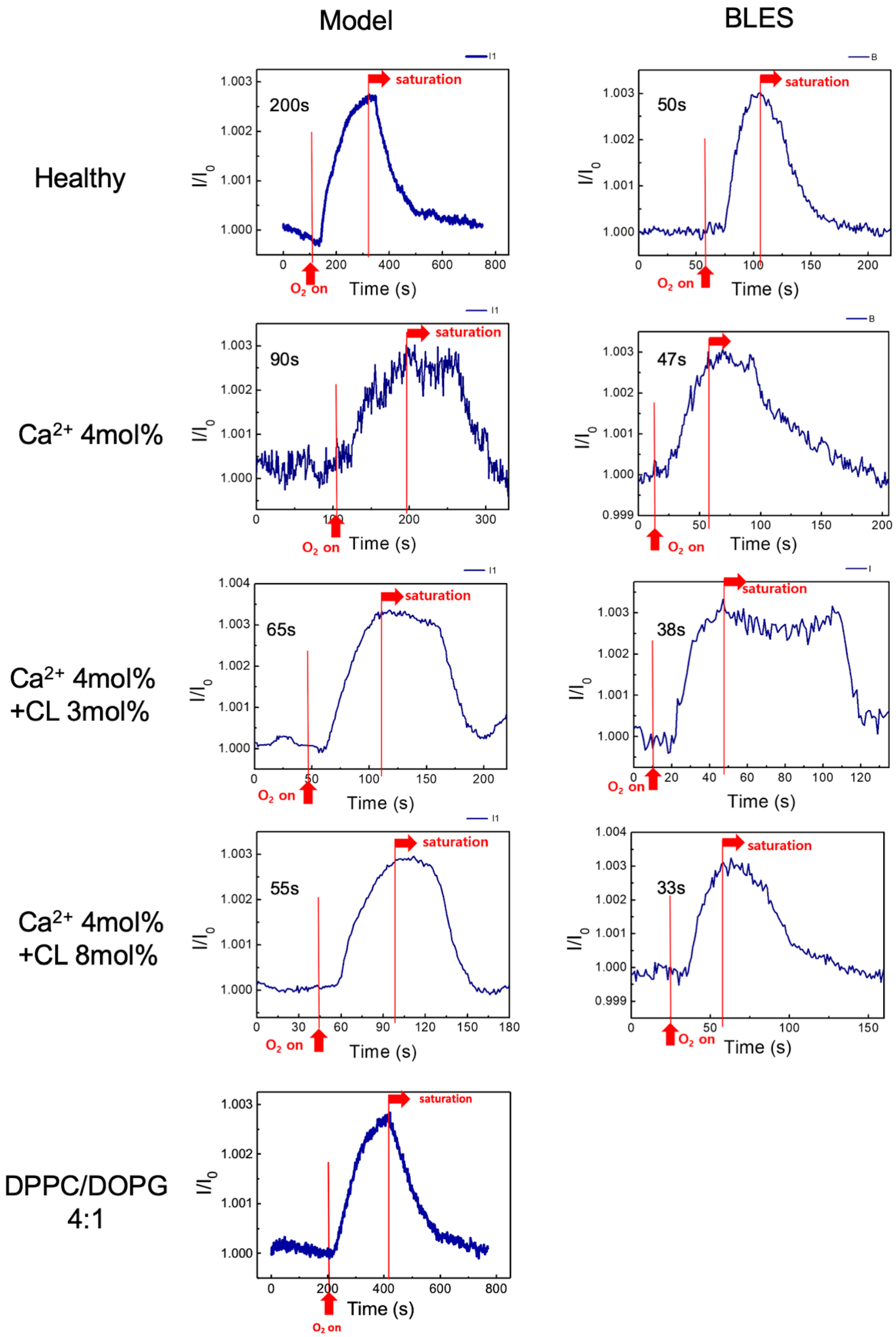

Supplementary Figure 6. FET gas sensor I/I0 measurements as a function of time (s) for model and BLES extract lung membranes at fixed Ca^{2+} concentration (4 mol%) and increasing CL.

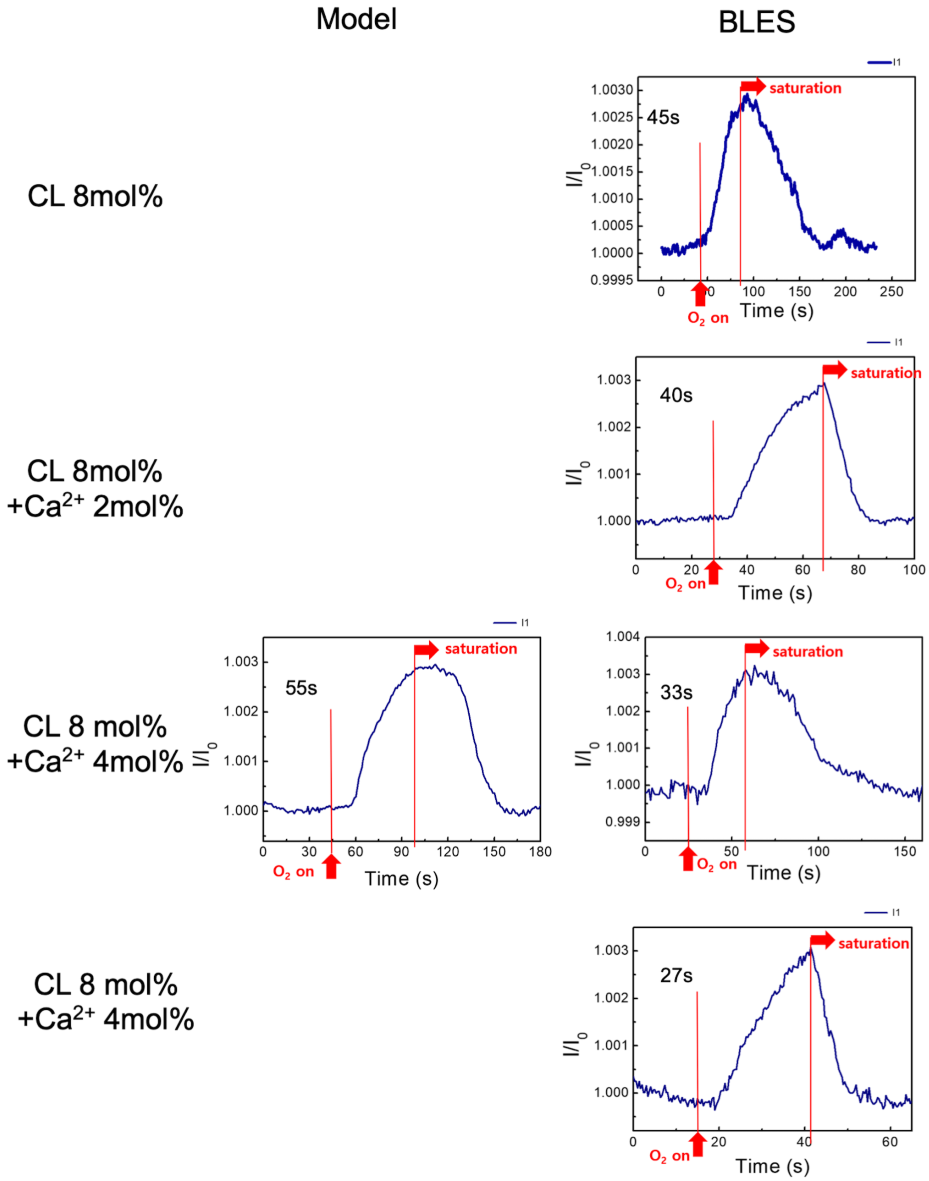

Supplementary Figure 7. FET gas sensor I/I0 measurements as a function of time (s) for model and BLES extract lung membranes at fixed CL concentration (8 mol%) and increasing Ca^{2+}.

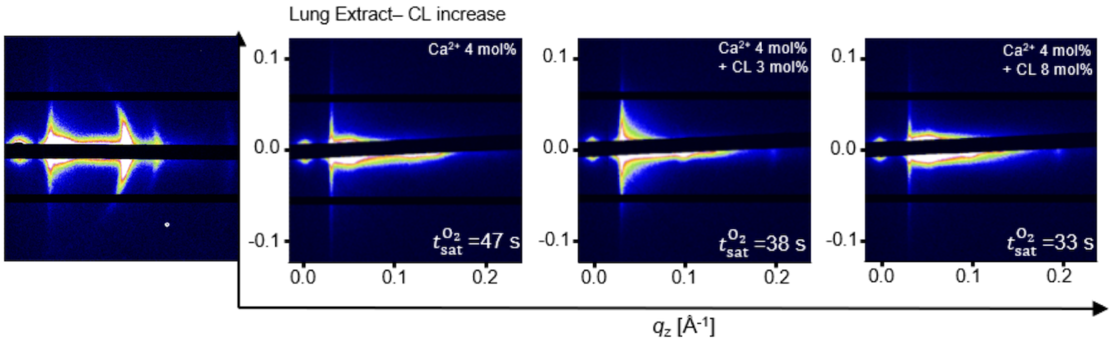

Supplementary Figure 8. 2D GISAXS data of BLES extract showing that a multilamellar arrangement is present with less orientational order compared to the lipid-only lung membrane model-system. As CL increases the orientation and lamellarity of the BLES films get significantly weaker. No stalk-phase diffraction peaks can be detected for any BLES sample.

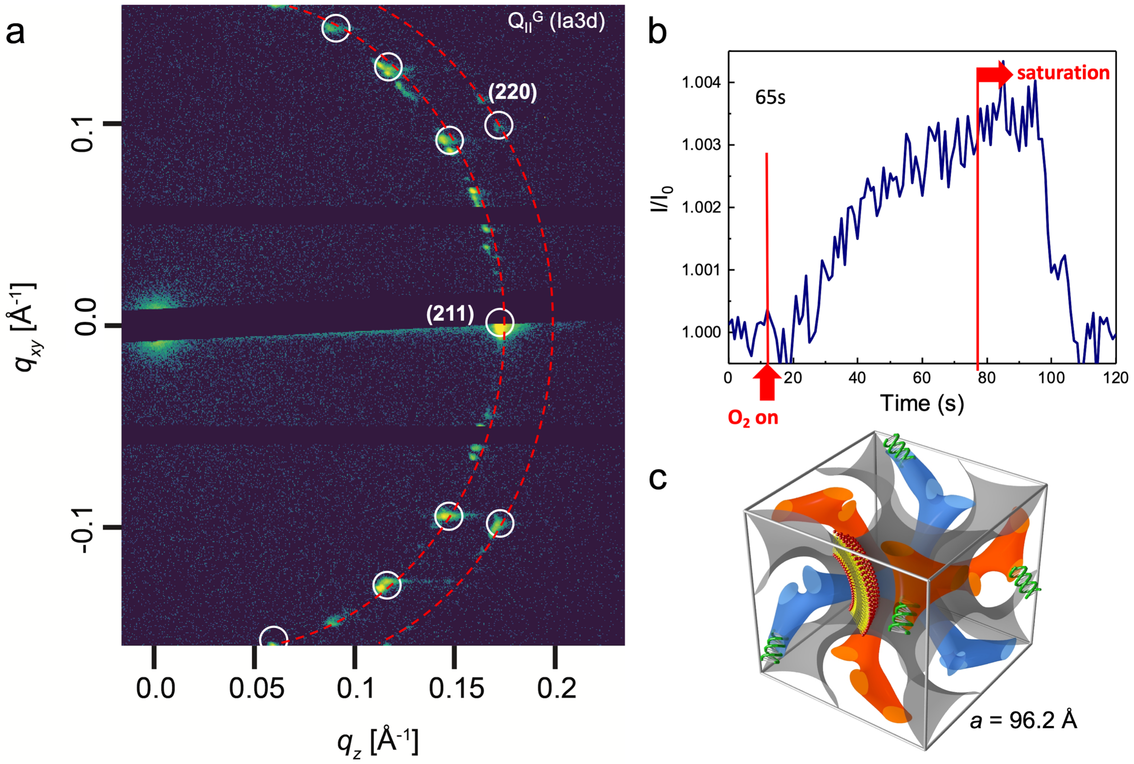

Supplementary Figure 9. Oxygen permeation through non-lamellar lipid structures. (a) In–house GISAXS diffraction patterns of a well oriented bicontinuous cubic phase (gyroid) formed by the composition of glycerol monooleate – GMO, and the univalent cationic lipid 1,2-dioleoyl-3-trimethylammonium-propane chloride salt – DOTAP at a molar ratio GMO/DOTAP 85:15 at $>$ 98% relative humidity at 37 °C. (b) Oxygen permeation measurement through the cubic phase film – saturation time of ($t_{sat}^{O_2}$) = 64 s. (c) Schematic representation of a bicontinuous cubic gyroid. The gray surface represents the midplane of the lipid bilayer that separates two water domains (orange and blue).

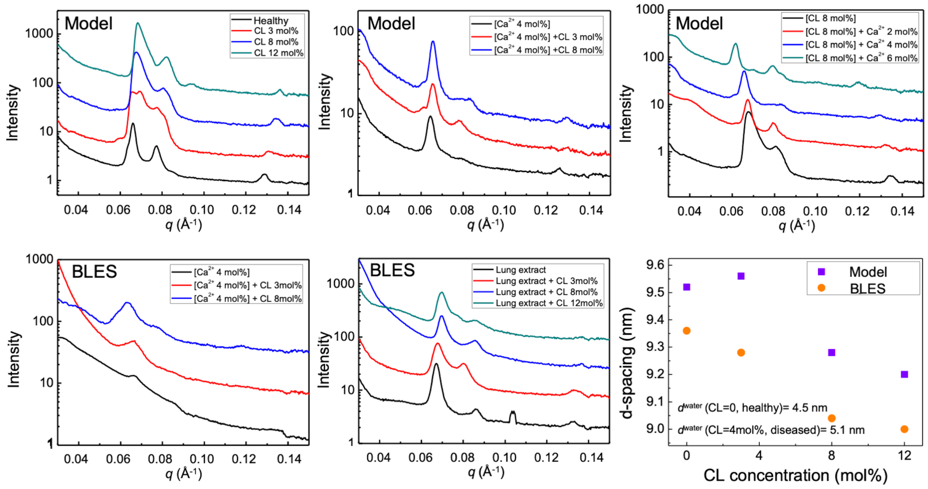

Supplementary Figure 10. Integrated GISAXS patterns for model and extract lung membrane systems as varied compositions. The repeat distance of the multilamellar phases decreases as a function of CL content as previously encountered for the same systems in bulk [1].The d–spacing measured by SAXS allows to estimate the thickness of the water layer as indicated in the figure assuming a normal bilayer thickness of 5 nm.

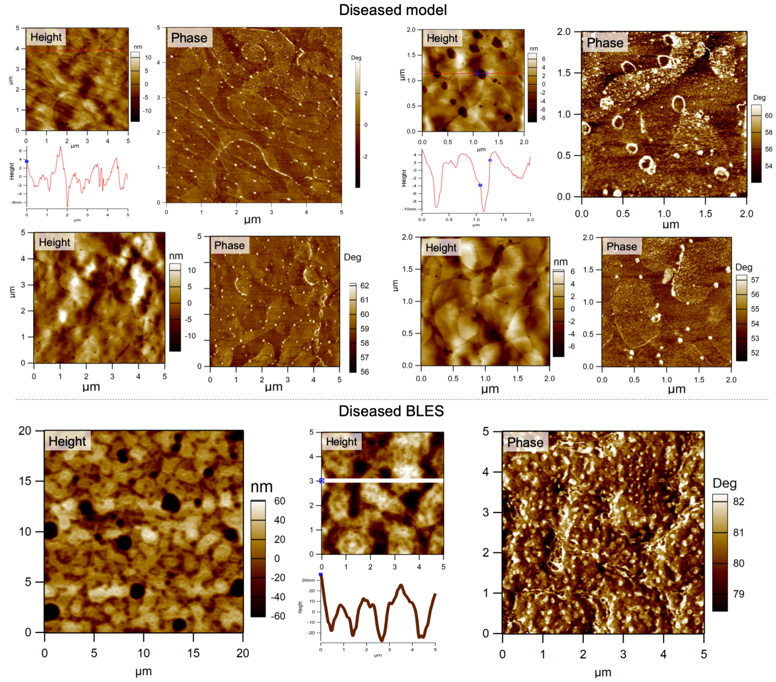

Supplementary Figure 11. A set of AFM height and phase images of the diseased model and BLES systems. The cross section profiles corresponding to the height variation along the lines drawn are shown below the top height images. The white dots visible in particular in the phase images correspond to the appearance of pore-like defects in the diseased model and BLES systems with Ca^{2+} and CL at high humidity conditions with size varying from a few nanometers to 50 nm.

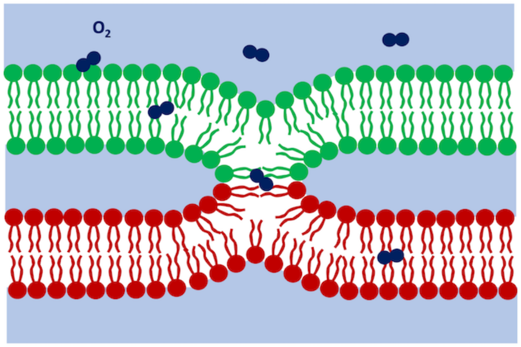

Supplementary Figure 12. Schematic representation of a inter-bilayer contact. The hemifusion region, or stalk, promotes transport of oxygen out of the alveoli hypophase into the blood stream.

References

[1] Steer, D., Leung, S. S. W., Meiselman, H., Topgaard, D. & Leal, C. Structure of lung-mimetic multilamellar bodies with lipid compositions relevant in pneumonia. *Langmuir* **34**, 7561–7574 (2018).
